# Balancing water and radiation productivity suggests a clue for improving yields in wheat under combined water deficit and terminal heat stress

**DOI:** 10.3389/fpls.2023.1171479

**Published:** 2023-05-16

**Authors:** Rajkumar Dhakar, Shivani Nagar, Vinay Kumar Sehgal, Prakash Kumar Jha, Madan Pal Singh, Debasish Chakraborty, Joydeep Mukherjee, P.V. Vara Prasad

**Affiliations:** ^1^ Division of Agricultural Physics, ICAR - Indian Agricultural Research Institute, New Delhi, India; ^2^ Division of Plant Physiology, ICAR - Indian Agricultural Research Institute, New Delhi, India; ^3^ Feed the Future Sustainable Intensification Innovation Lab, Department of Agronomy, Kansas State University, Manhattan, KS, United States; ^4^ Department of Agronomy, Kansas State University, Manhattan, KS, United States

**Keywords:** canopy conductance, water use, light interception, water use efficiency, radiation use efficiency

## Abstract

Sustaining crop yield under abiotic stresses with optimized resource use is a prerequisite for sustainable agriculture, especially in arid and semi-arid areas. Water and heat stress are major abiotic stresses impacting crop growth and yield by influencing complex physiological and biochemical processes during the life cycle of crops. In a 2-year (2015–2017) research, spring wheat cv. HD-2967 was grown under deficit irrigation and delayed sowing conditions to impose water and terminal heat stresses, respectively. The data were analyzed for seasonal crop water use, radiation interception, water productivity (WP), and radiation productivity (RP) under combined water deficit and terminal heat stresses. Seasonal crop water use was significantly affected by stresses in the order of water + terminal heat > water > terminal heat. Water stress showed minimal effect on the light extinction coefficient and consequently on seasonal intercepted photosynthetically active radiation (IPAR). However, seasonal IPAR was primarily affected by combined water + terminal heat and terminal heat stress alone. The slope of crop water use and IPAR, i.e., canopy conductance, an indicator of canopy stomatal conductance, was more influenced by water stress than by terminal heat stress. Results showed that linear proportionality between WP and RP is no longer valid under stress conditions, as it follows a curvilinear relation. This is further supported by the fact that independent productivity (either water or radiation) lacked the ability to explain variability in the final economic yield or biomass of wheat. However, the ratio of RP to WP explained the variability in wheat yield/biomass under individual or combined stresses. This suggests a clue for improving higher wheat yield under stress by managing WP and RP. The highest biomass or yield is realized when the ratio of RP to WP approaches unity. Screening of genotypes for traits leading to a higher ratio of RP to WP provides an opportunity for improving wheat productivity under stressed environments.

## 1 Introduction

The increasing population in the global south faces the challenge of enhancing food production from limited available resources. Water is increasingly becoming a limited resource in recent years due to the overexploitation of groundwater and increased evapotranspiration in the wake of climate change. Recent reports have reported that the extent of productive cropping systems in Asia has been receiving lesser sunlight in recent years as compared to the past due to increased aerosols in the atmosphere (van Oldenborgh et al., 2018). Resources like water and radiation may pose a serious constraint to agricultural production and its sustainability in the near future. One of the effective strategies for sustainable agricultural production is to enhance the efficiencies or productivity of resources like water, radiation, and nutrients. In the past few decades, targeted efforts are being made by researchers to understand the water productivity (WP) and radiation productivity (RP) of various crops under different environments in relation to crop productivity (Caviglia and Sadras, 2001; Narayanan et al., 2013; Camargo et al., 2016; Pradhan et al., 2018).

Wheat (*Triticum aestivum* L.) ranks second in global food grain production and consumption. However, its productivity is threatened by mainly abiotic stresses like drought and heat (Prasad et al., 2008b; Prasad et al., 2011; Sehgal et al., 2018; Priya et al., 2019). Both drought and heat are associated with each other and often occur in combination, and these stresses will further increase due to climate change and variability. High temperature, especially during the reproductive stage leading to terminal heat stress in wheat, affects grain development, grain filling duration, and the source–sink relationship, consequently lowering grain numbers, individual grain weights, and final yield (Prasad et al., 2008b; Prasad et al., 2011; Reynolds et al., 2012; Mondal et al., 2013; Nagar et al., 2015b; Yadav et al., 2022). Water deficit stress hampers growth, development, nutrient and water relations, and photo-assimilation and its partitioning, ultimately leading to a decline in crop yields (Farooq et al., 2011; Nagar et al., 2015a; Jha et al., 2018). However, the interactive effect of water deficit and heat stress is complex in nature and studies about them are less common (Prasad et al., 2008a; Pradhan et al., 2012a; Sehgal et al., 2017; Hlaváčová et al., 2018; Sehgal et al., 2018; Hussain et al., 2019). Most of these studies emphasized the effect of combined stress on physiological processes such as oxidative stress, photosynthesis, yield attributes, and yield. However, there is limited knowledge on the effect of drought and heat stresses on light interception and crop water use, an important determinant of aboveground biomass (AGB) and crop yield.

Productivity is generally expressed as a quotient of output and input. The output is taken as biomass while input may be taken as water transpired or the amount of radiation intercepted by the canopy over its growing period. The concept of WP and RP is widely used in crop growth models (Monteith, 1977; Sinclair and Muchow, 1999). Significant variation in WP and RP of crop plants has been reported among the species as well as among genotypes within species. Both are affected by environmental factors and management practices. Water stress affects WP through both stomatal and non-stomatal limitations (Martin and Ruiz-Torres, 1992). Moderate water stress generally increases the WP of plants by lowering the transpiration rate through reduced stomatal aperture (Van den Boogaard et al., 1997; Rekika et al., 1998). However, under conditions of severe water stress, a decrease in WP has been reported in plants, which are usually associated with a larger decrease in photosynthetic rate due to impaired biochemical, physiological, or metabolic changes (El Hafid et al., 1998; Anyia and Herzog, 2004; Jha et al., 2018). Water stress may be associated with an increase in WP; however, it frequently leads to reduced yield (Blum, 2005). Water stress causes a reduction in leaf area index (LAI), which consequently results in reduced intercepted photosynthetically active radiation (IPAR), ultimately lowering the RP (O’Connell et al., 2004). RP is a function of the light extinction coefficient (*k*) that determines the efficiency of light interception by crop canopies. Several authors have shown that deficit irrigation had a non-significant effect on *k* of wheat in semi-arid locations (Thomas, 2013; Pradhan et al., 2018). Temperature is another important meteorological factor that influences the WP and RP. Mendham et al. (1981) showed that oilseed crops exposed to high temperatures due to delayed sowing resulted in lower RUE than normal sown crops. Pandey et al. (2004) reported a decrease in RP with delayed sowing of wheat at Anand in India. However, there is limited information on the effect of terminal heat stress on *k*, WP, and RP in wheat crops altogether.

The understanding of either WP or RP in various crops has been done independently, and limited studies that explore the link between the two are available. Moreover, few studies have investigated such relations under deficit water (Caviglia and Sadras, 2001; Narayanan et al., 2013). In general, WP and RP are linearly proportional to each other, and the proportionality constant is termed “crop conductance”, representing the amount of crop water used or transpired per unit of intercepted radiation (Sadras et al., 1991; Caviglia and Sadras, 2001). An important unanswered question is: Does the linear proportionality of WP and RP hold true under stressed conditions? To answer this, the current study was designed with the following objectives: (i) to understand the interactive effect of water deficit and terminal heat stress on crop water use, IPAR, and biomass production of spring wheat grown in a semi-arid environment; (ii) to investigate the effect of water deficit and terminal heat stress on resource productivity of the wheat crop at the field scale; (iii) to examine the proportionality of WP and RP in spring wheat under stressed conditions; and (iv) to test the hypothesis of whether a balance between WP and RP may lead to higher wheat yield under stressed conditions. This study will advance the knowledge of enhancing wheat productivity under stressed environments.

## 2 Materials and methods

### 2.1 Study area and experimental details

A field experiment was conducted at the experimental farm (Main Block 4C) of the Division of Agricultural Physics, Indian Agricultural Research Institute, New Delhi, located at 28°38′23″N latitude and 77°09′27″E longitude with an altitude of 228.6 m above mean sea level. The climate was subtropical and semi-arid characterized by a hot–dry summer and a cold winter. The mean monthly maximum temperature in the *rabi* season (November to April) ranges from 20 to 36°C and the mean monthly minimum temperature ranges from 6 to 19°C. The mean annual rainfall (30 years average) was 769.3 mm, of which 75% is received during the southwest monsoon season between July and September, and the remaining rain is received in the *rabi* season.

The extent of the experimental site was 0.055 ha, out of which the net sown area was 0.036 ha with an individual plot size of 5 m by 5 m. The soils of the site are deep, well-drained, and sandy loam in texture throughout the profile. Spring wheat (cv. HD-2967) was grown during the *rabi* seasons of 2015–2016 and 2016–2017 in a split-plot design with the number of irrigations as main treatments and date of sowing as subplot treatments with three replications. The field was prepared following the usual pre-sowing operations of disking and leveling. The irrigation treatments included the following: I5, five irrigations [at crown root initiation (CRI), tillering, booting, flowering, and milking stages]; I3, three irrigations (at CRI, tillering, and flowering stages); and I1, one irrigation (at CRI stage). Approximately 60 mm of water was applied in each irrigation event, as measured by Parshall Flume. The two dates of sowing treatments were as follows: D_1_, timely sown (20 November 2015) and D_2_, late sown (9 December 2015) during *rabi* season 2015–2016; and D_1_, timely sown (17 November 2016) and D_2_, late sown (7 December 2016) during 2016–2017. The sowing was done manually using a handheld seed drill with the recommended spacing of 22.5 cm between rows. A plant–plant distance of 5 cm was followed as practice in the wheat belt of Indo-Gangetic plains. The recommended dose of NPK fertilizers, i.e., 120:60:60 was applied. Urea as nitrogenous fertilizer was applied in three doses (50% as basal during sowing, 25% during the CRI stage, and 25% during the flowering stage). However, in the case of I1 treatment, urea was applied in two doses (50% as pre-plant incorporation and 50% during the CRI stage) synchronizing with irrigation dates. The whole amount of P and K was applied in a single dose during sowing. The recommended cultural practices of weeding and plant protection measures were followed. The crop was hand-harvested after complete drying.

### 2.2 Aboveground biomass

Dry AGB was measured periodically during crop growth using destructive sampling. AGB and crop yield at harvest were measured on a unit area (per m^2^) basis and expressed as kg ha^−1^.

### 2.3 Intercepted photosynthetically active radiation

Line Quantum Sensor (LI-191, LI-COR Biosciences, Lincoln, NE, USA) was used with an integrator (LI- 250A, LI-COR Bioscience, Lincoln, NE, USA) for measuring the incident and IPAR by a wheat canopy. PAR measurements were taken above the canopy with the sensor facing the sky to account for incident radiation (*I*_o_) received and the sensor looking downwards for reflected radiation (*I*_r_) from the canopy. Data were recorded below the canopy keeping the sensor just above the soil but across the rows with the sensor looking upwards for the transmitted radiation (*I*_t_) through the canopy and with the sensor looking downwards for radiation reflected (*I*_e_) from the soil. Three sets of measurements were recorded in each plot and averaged. The above measurements were taken at regular intervals on clear days between 11:30 and 12:00 hours IST (Indian Standard Time) when disturbances due to leaf shading and solar angle were minimum. These measurements were used to derive the fraction IPAR (*f*IPAR) as given in the formula:


(1) $$fIPAR=\frac{\left(I_{0}-I_{t}\right)}{I_{t}}$$

Values for *f*IPAR for each day after sowing were interpolated between actual measurements by linear interpolation throughout the crop season. Daily bright sunshine hours (*n*) values were recorded at the meteorological observatory and were used to calculate solar radiation (MJ m^−2^) by using the Angstrom formula (using coefficients *a* = 0.32, *b* = 0.46).


(2) Solar radiation=Ra*(a+(b*nN))

where *N* is astronomically maximum possible sunshine hours and *R_a_* is extraterrestrial solar radiation.

The daily incident PAR was calculated by multiplying global radiation with a factor of 0.48. Daily incoming PAR values were multiplied by corresponding daily *f*IPAR values to compute daily IPAR. The daily IPAR was accumulated corresponding to the crop growth period.

### 2.4 Crop water use

Crop water use was calculated using the field water balance equation; i.e., crop water use is equal to the sum of water applied as irrigation, rainfall received, and soil moisture storage applied. Soil moisture content (v/v, %) up to 120 cm depth was measured periodically using Neutron Moisture Probe (CPN-503 DR Hydroprobe, Campbell Pacific Nuclear International Inc. USA). Soil moisture storage was computed from the depletion of moisture from 0 to 120 cm soil depth between two subsequent dates, assuming no deep drainage occurred, no capillary rise contribution from the water table as it is quite deep (>10 m), and no runoff occurred as the plots were bunded to a height of 15 cm.

### 2.5 Canopy conductance, water productivity, and radiation productivity

The canopy conductance (CC) (mm-m^2^ MJ^−1^) was determined with least-square regression by calculating the slope of the regression between crop water use (mm) and IPAR (MJ m^−2^) with an intercept set to zero. WP (g m^−2^ mm^−1^) was calculated as the slope of the linear regression between AGB (g m^−2^) and cumulative crop water use (mm) by keeping the intercept as zero. RP (g MJ^−1^) was calculated as the slope of the linear regression between AGB (g m^−2^) and cumulative total IPAR (MJ m^−2^) by keeping the intercept as zero.

### 2.6 Statistical analyses

Analysis of variance (ANOVA) as applicable to split plot design was performed using the “aov” function available in the “stats” package of statistical software “R”. Tukey’s “Honest Significant Difference” method (using the TukeyHSD function in “R”) was used to analyze the differences between the means of different factors at a 5% probability level. Box-and-whisker plots of the response variable were created using the “boxplot” function available in the “graphics” package of R. Regression analyses were performed using the data analysis tool pack of MS Excel (2013) and statistical significance of coefficient of determination (*R* ^2^) was tested through ANOVA.

## 3 Results

### 3.1 Environmental conditions

**Figure 1:**
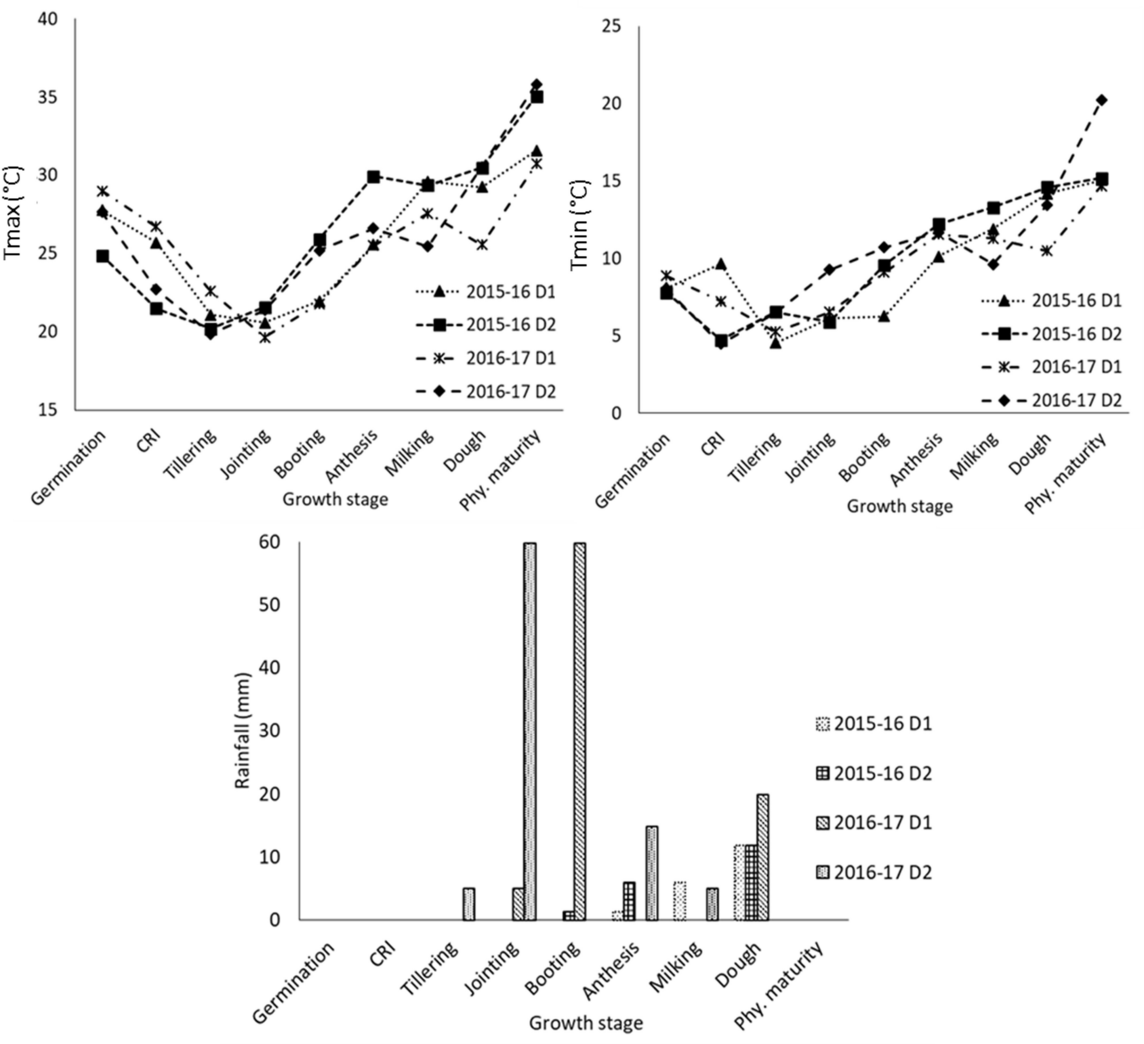
Meteorological conditions during the crop growth at different growth stages in 2015-16 and 2016-17 under different sowing dates (D1 normal; and D2 delayed sowing). CRI, crown root initiation; Phy. maturity, physiological maturity.

The phenophase-wise mean of maximum and minimum temperature and rainfall for two sowing dates in both years, i.e., 2015–2016 and 2016–2017, are presented in **Figure 1**. It shows that higher maximum temperatures prevailed during the reproductive stage (~4°C higher during booting to anthesis and dough to physiological maturity) for delayed sowing compared to normal sowing in 2015–2016. In 2016–2017, maximum temperatures were higher by ~2.5–5°C in delayed sown crops compared to that of the normal sown crop during jointing to booting and milking to physiological maturity stages. In both years, the mean maximum temperature was almost similar during the dough to physiological maturity stage in delayed sown crops, but minimum temperatures were higher during 2016–2017. In normally sown crops, the mean maximum temperature during anthesis to physiological maturity was higher (1–2°C) in 2015–2016 than that in 2016–2017. Thus, delayed sown crops experienced relatively higher temperatures, especially during reproductive stages, and experienced heat stress in both years compared to normal sowing. The wheat growing season of 2016–2017 received more rainfall (87.4 mm) than that of the previous year (19.2 mm). In the year 2016–2017, the occurrence of a heavy rainfall event of 59.8 mm coincided with the jointing to the booting stage of the normal sown crop and tillering to the jointing stage of the delayed sown crop. Delayed sown crop in 2015–2016 received two significant rainfall events of 6 mm and 11.8 mm, coinciding with the anthesis and dough stages, respectively.

### 3.2 Canopy light extinction coefficient (k)

**Figure 2:**
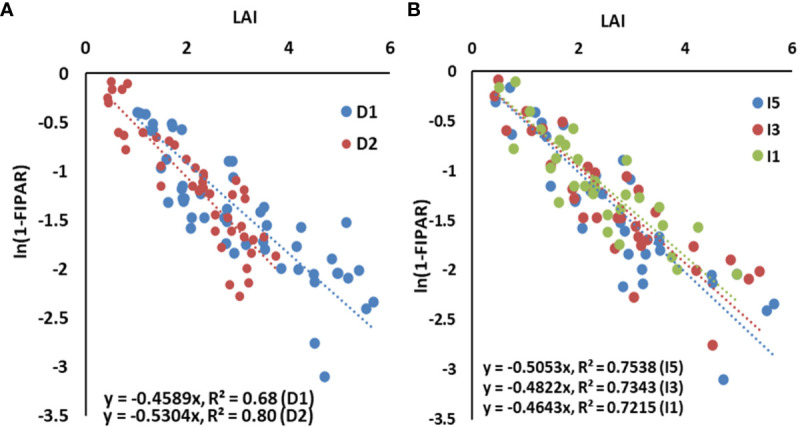
The light extinction coefficient of the wheat canopy as influenced by **(A)** sowing dates and **(B)** irrigation treatments, pooled for two years. LAI, leaf area index; IPAR, intercepted photosynthetically active radiation. Dl, normal sowing; D2 delayed sowing; 15, five irrigations; 13 three irrigations, and 11, one irrigation.

The slope of the regression line between LAI and ln(1 − fIPAR), i.e., canopy light extinction coefficient (*k*), was calculated to study the effect of sowing (heat stress) and irrigation treatments (water) on radiation interception, and results are shown in **Figure 2**. The degree of relationship as exhibited by *R* ^2^ varied between 0.68 and 0.80, which are statistically significant at *p*< 0.05. The “*k*” varied between 0.43 (D1I1) and 0.56 (D2I5) across the 2 years (data not shown) with a mean value of 0.50. The irrigation levels had a non-significant effect on the canopy light extinction coefficient. The estimated value of “*k*” was 0.50, 0.48, and 0.46 for I5, I3, and I1 treatments, respectively. Delay in sowing altered “*k*” from 0.46 in D1 to 0.53 in D2 sown crop, which is statistically significant.

### 3.3 Seasonal crop water use and IPAR

**Figure 3:**
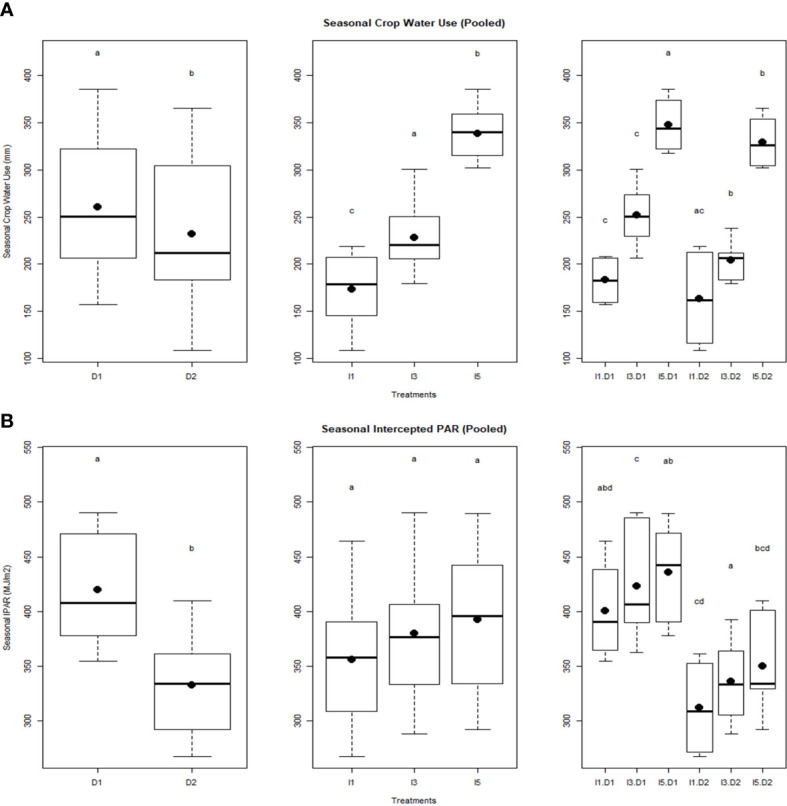
Effect of delayed sowing and irrigations on **(A)** Seasonal crop water use, and **(B)** Seasonal intercepted PAR. Letters over box and whisker plots indicate the significant differences in mean values through TukeyHSD test. D1, normal sowing; D2 delayed sowing; I5, five irrigations; I3 three irrigations, and I1, one irrigation.

Wheat crops exhibited significant variations in seasonal crop water use among the late sown and irrigation treatments (**Figure 3A**). A significant decrease in seasonal crop water use was noticed in the D2I1 treatment (170 mm) compared to the D1I5 treatment (330 mm). The increase in irrigation increased the seasonal crop water use. PAR intercepted during the crop growth period was significantly decreased by delayed planting, while it increased with the level of irrigation, but statistically, it was non-significant (**Figure 3B**). The interaction of delayed sowing and irrigation treatments also significantly affected the PAR interception. Thus, irrigation alone did not affect the total interception of PAR. Delayed planting reduced the seasonal IPAR by 21% over the normal sowing. The mean values of seasonal IPAR varied between 430 MJ m^−2^ (D1I5) and 320 MJ m^−2^ (D2I1) in the 2 years of experimentation.

### 3.4 Biomass production

**Figure 4:**
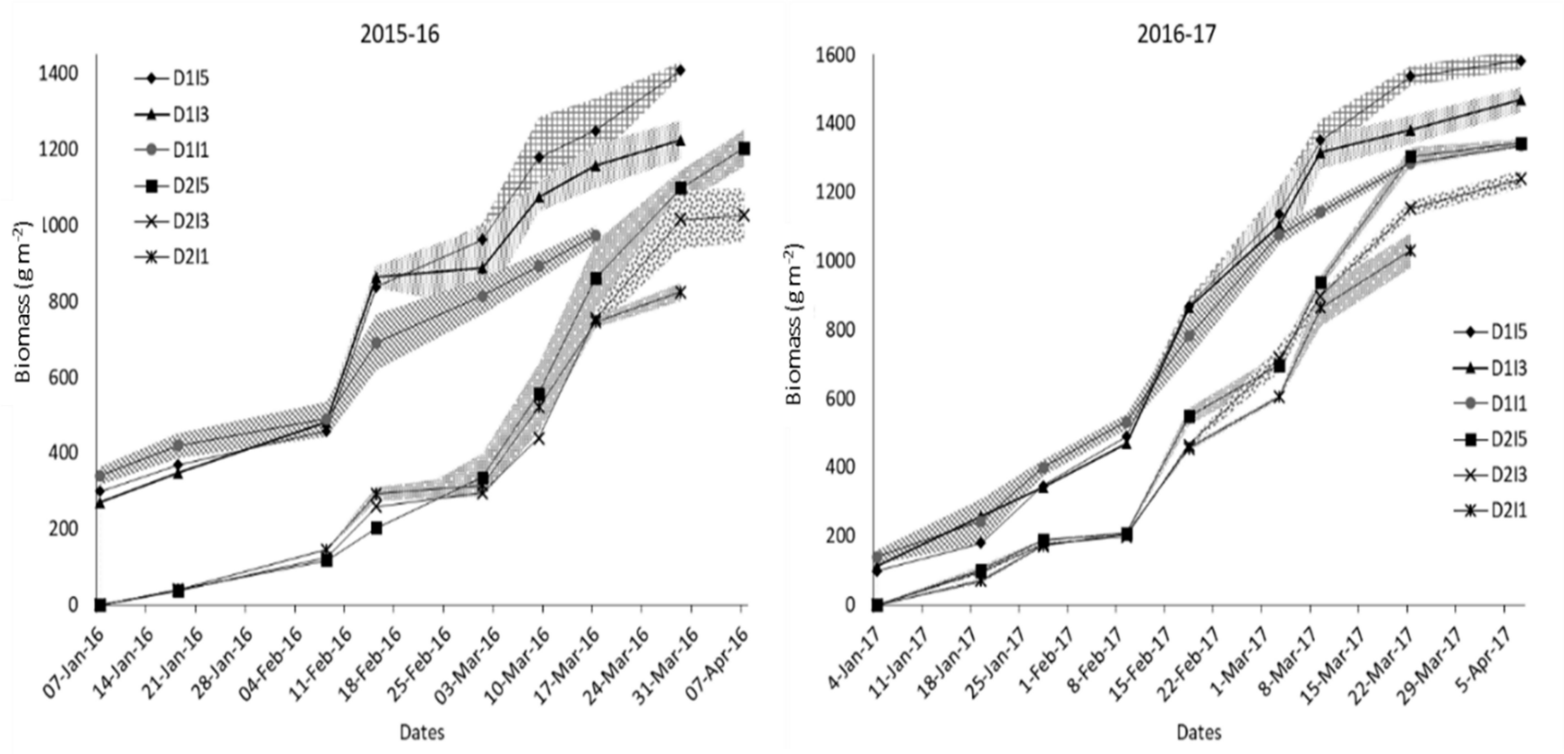
Temporal profile of biomass production for different sowing and irrigation levels. The shaded area shows the standard error of the observation. D1, normal sowing; D2 delayed sowing; I5, five irrigations; I3 three irrigations, and I1, one irrigation.

**Figure 5:**
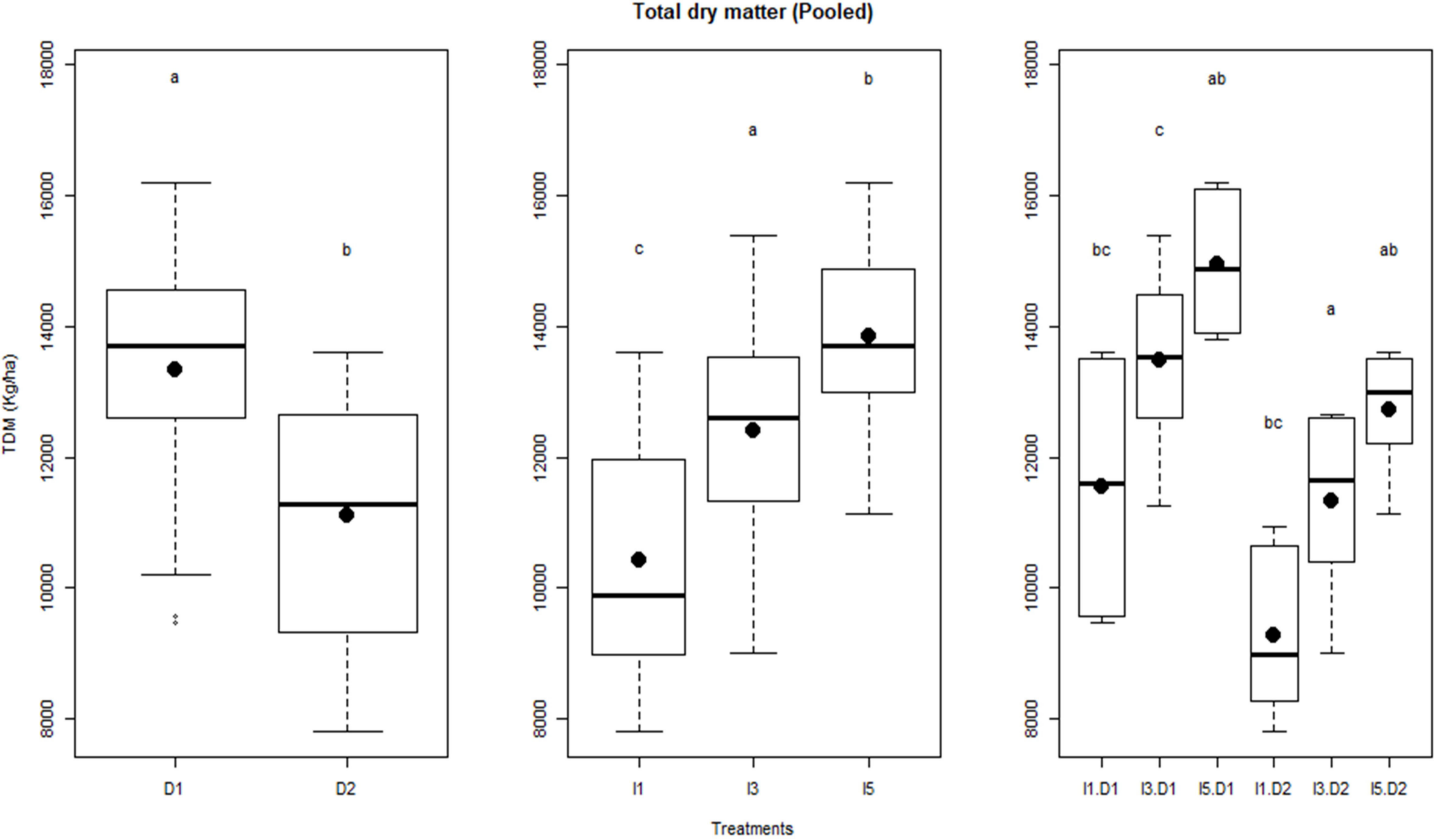
Influence of delayed sowing and irrigations on total dry matter (TDM) production of wheat pooled over the two years. Letters over box and whisker plots indicate the significant differences in mean values through TukeyHSD test. D1, normal sowing; D2 delayed sowing; I5, five irrigations; I3 three irrigations, and I1, one irrigation.

The temporal variation in biomass for sowing dates and irrigation treatments over the 2 years is shown in **Figure 4**. Crops started differing in biomass production after 50 and 35 days after sowing in normal and late sowing, respectively. In delayed sowing, significant differences in biomass production were observed only after anthesis, where I5 and I3 treatments had higher biomass than the I1 treatment during the rest of the growth period in 2015–2016. Irrigation treatments had a little differential effect on biomass production between the sowing and the anthesis stage of crop growth in both years. Pooled analysis showed that irrigation treatments, delayed sowing, and their interactions significantly influenced final dry biomass (**Figure 5**).

### 3.5 Water productivity

**Figure 6:**
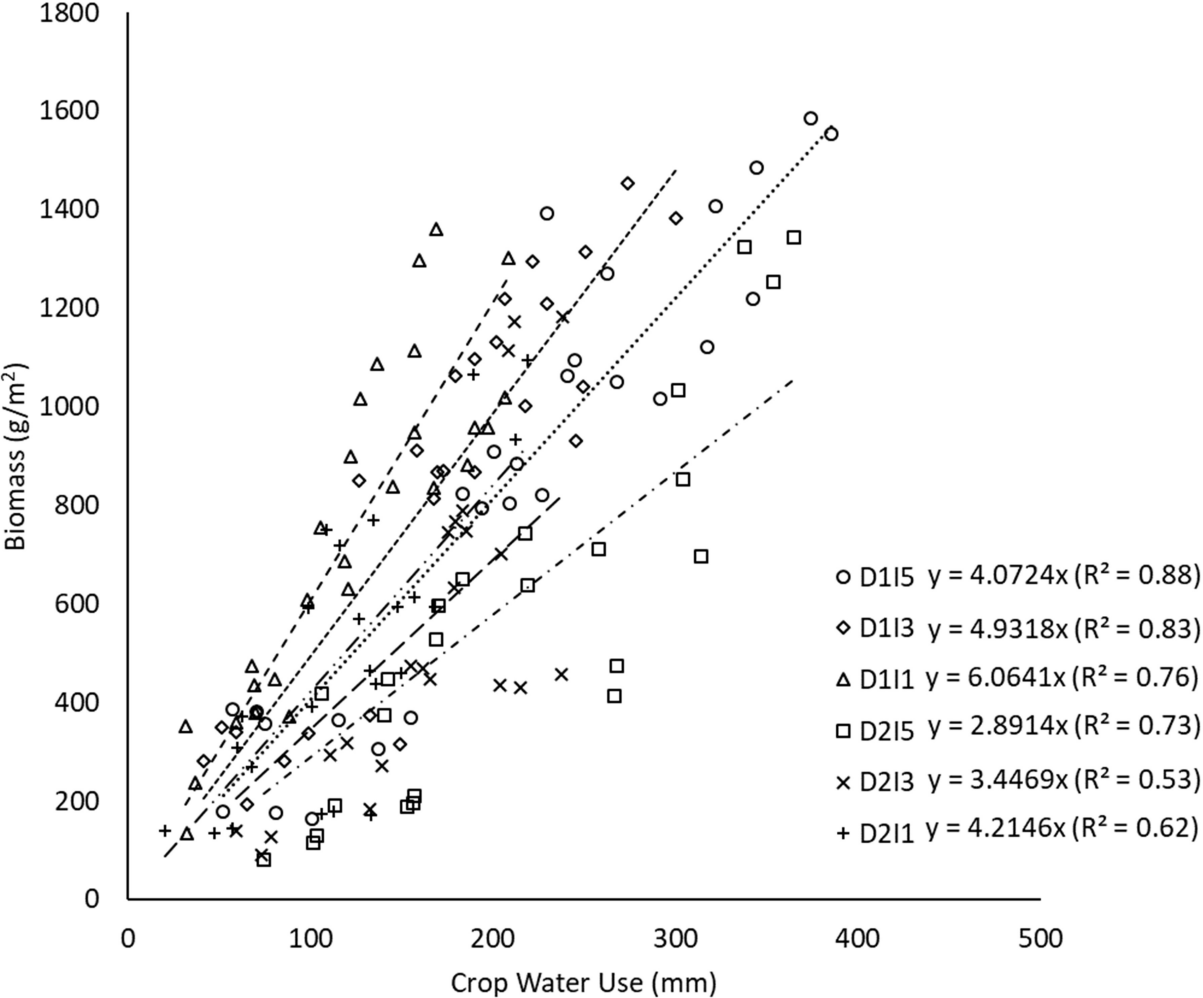
Regression between biomass and cumulative crop water use under different sowing and irrigation treatments (slope indicates WP) for data pooled over the two years. D1, normal sowing; D2 delayed sowing; I5, five irrigations; I3 three irrigations, and I1, one irrigation.

**Figure 7:**
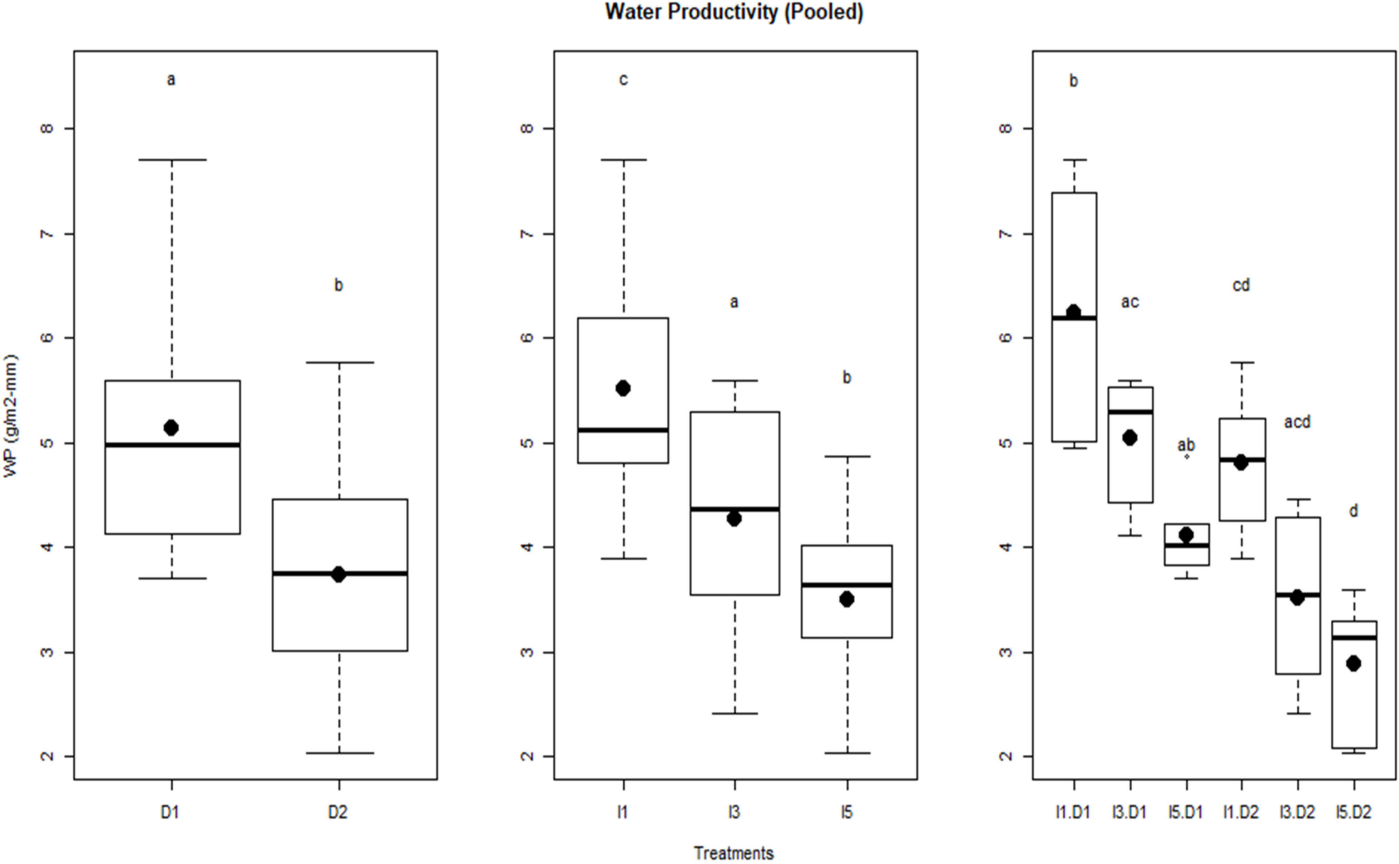
Water productivity of wheat as influenced by sowing and irrigation treatments for pooled data over the two years. Letters over box and whisker plots indicate the significant differences in mean values through TukeyHSD test. D1, normal sowing; D2 delayed sowing; I5, five irrigations; I3 three irrigations, and I1, one irrigation.

The linear regression between biomass and cumulative crop water use during crop growth under different sowing and irrigation treatments is shown in **Figure 6**. The slope of the regression line represents the WP in each case. The WP (i.e., biomass produced per unit of crop water use) varied between 2.9 (D2I5) and 6.0 g/m^2^-mm for the pooled data over the 2 years (**Figure 6**). **Figure 6** also shows that the strength of the linear relationship (in terms of *R* ^2^) successively reduced as the environment became stressful (delayed sowing with deficit irrigation). An opposite pattern was seen in WP. **Figure 7** shows the effect of sowing dates and irrigation treatments on seasonal WP. It indicates significant differences (*p*< 0.05) in WP when plants are exposed to temperature stress through delayed sowing. Delayed sowing decreased WP by approximately 27%.

It may be due to greater evapotranspiration (ET) caused by progressively higher vapor pressure deficit (associated with higher temperature) experienced by the crop during the later stage of the crop season. Irrigation treatments significantly affected WP; reduced water availability resulted in the enhancement in WP. The I1 treatment (least irrigation) had the highest WP (5.5 g/m^2^-mm) followed by the I3 treatment (4.2 g/m^2^-mm) and the lowest was observed in the I5 treatment (3.5–4.2 g/m^2^-mm). Significant differences in WP were observed in the interaction of irrigation and delayed sowing treatments. Results indicate that crops sown at a normal time (D1 sowing) and with one irrigation efficiently used the available water resulting in higher WP compared to other treatments. It also shows that the D1I1 treatment had greater variability in WP measurements over 2 years of study than any other treatment.

### 3.6 Radiation productivity

**Figure 8:**
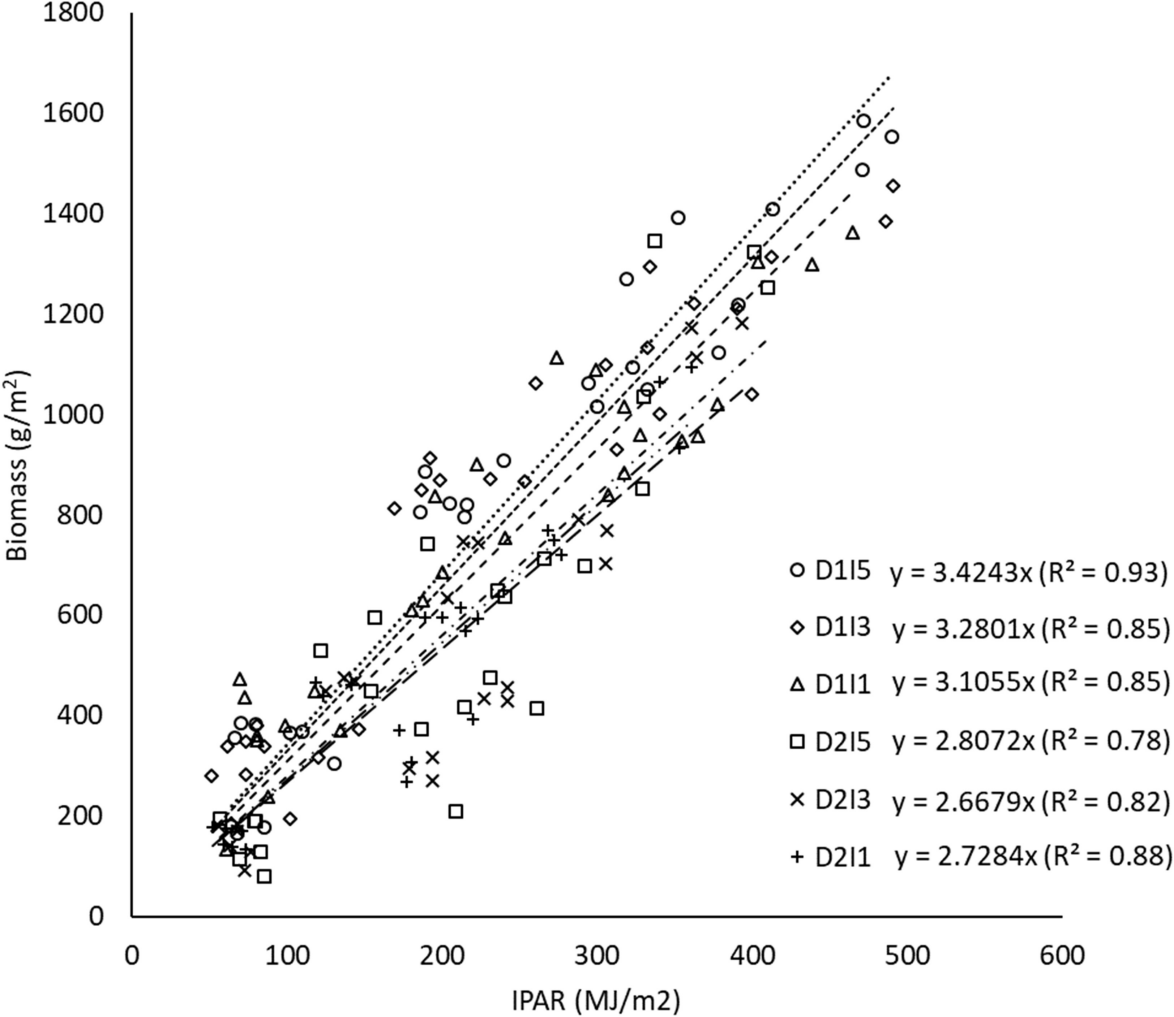
Regression between biomass and cumulative intercepted PAR for different sowing and inigation treatments (the slope indicates RP) for data pooled over the two years. D1, normal sowing; D2 delayed sowing; I5, five irrigations; I3 three irrigations, and I1, one irrigation.

**Figure 9:**
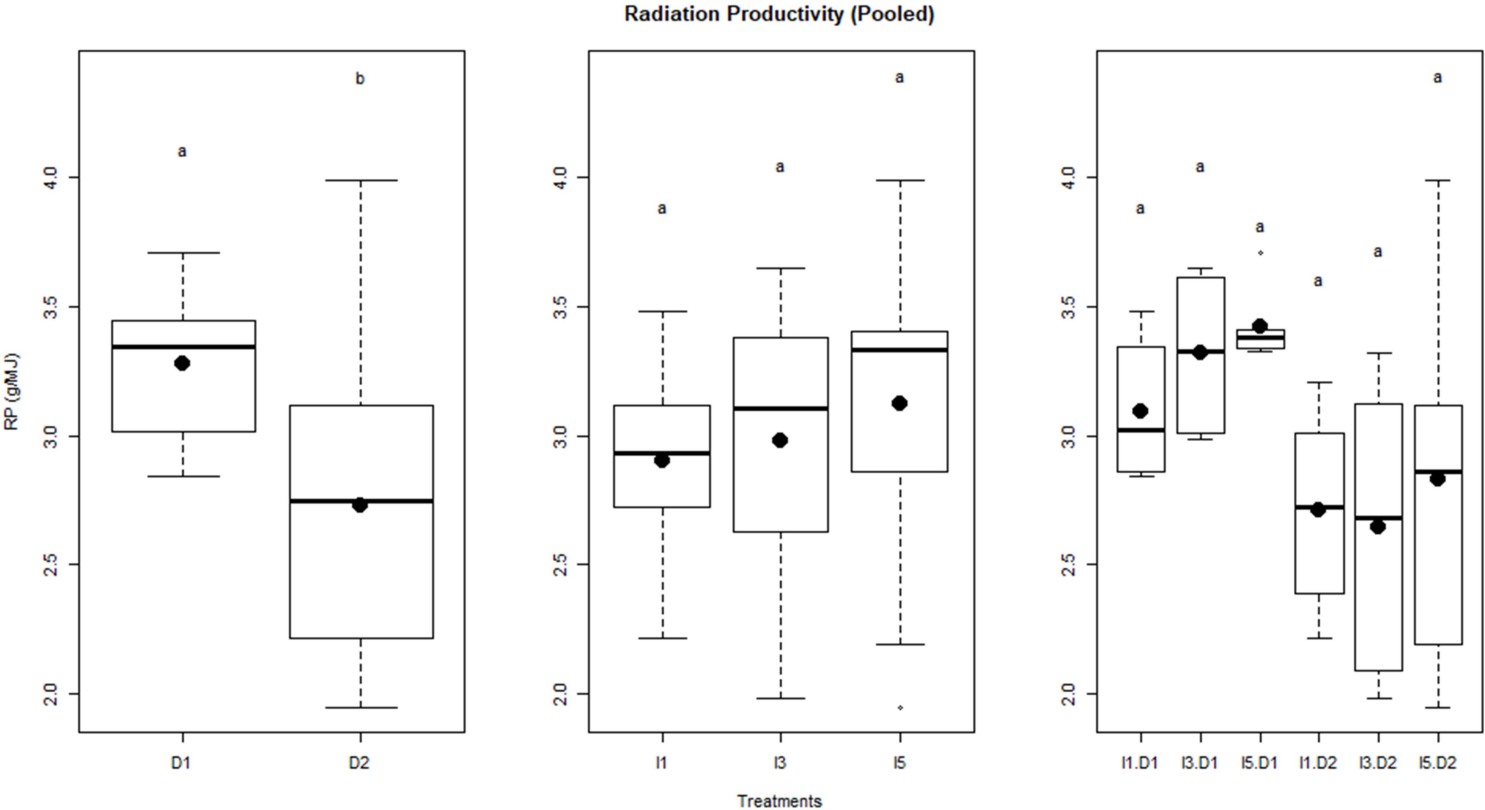
Radiation productivity of wheat as influenced by sowing and irrigation treatments. Letters over box and whisker plots indicate the significant differences in mean values through TukeyHSD test. Dl, normal sowing; D2 delayed sowing; I5, five irrigations; I3 three irrigations, and I1, one irrigation.

The relationship between biomass and cumulative IPAR during crop growth is shown as least-square regression lines for different treatments (**Figure 8**). The slope of least-square regression lines represents the RP of the crop, i.e., biomass per unit of cumulative IPAR. The RP of wheat varied between 2.66 g MJ^−1^ and 3.42 g MJ^−1^ for the pooled data over the 2 years, i.e., 2015–2016 and 2016–2017. It was observed that delay in sowing caused a significant reduction in RP by ~16% (**Figure 9**). However, RP values were not significantly different among irrigation treatments and the interaction of sowing date and irrigation treatments.

### 3.7 Canopy conductance

**Figure 10:**
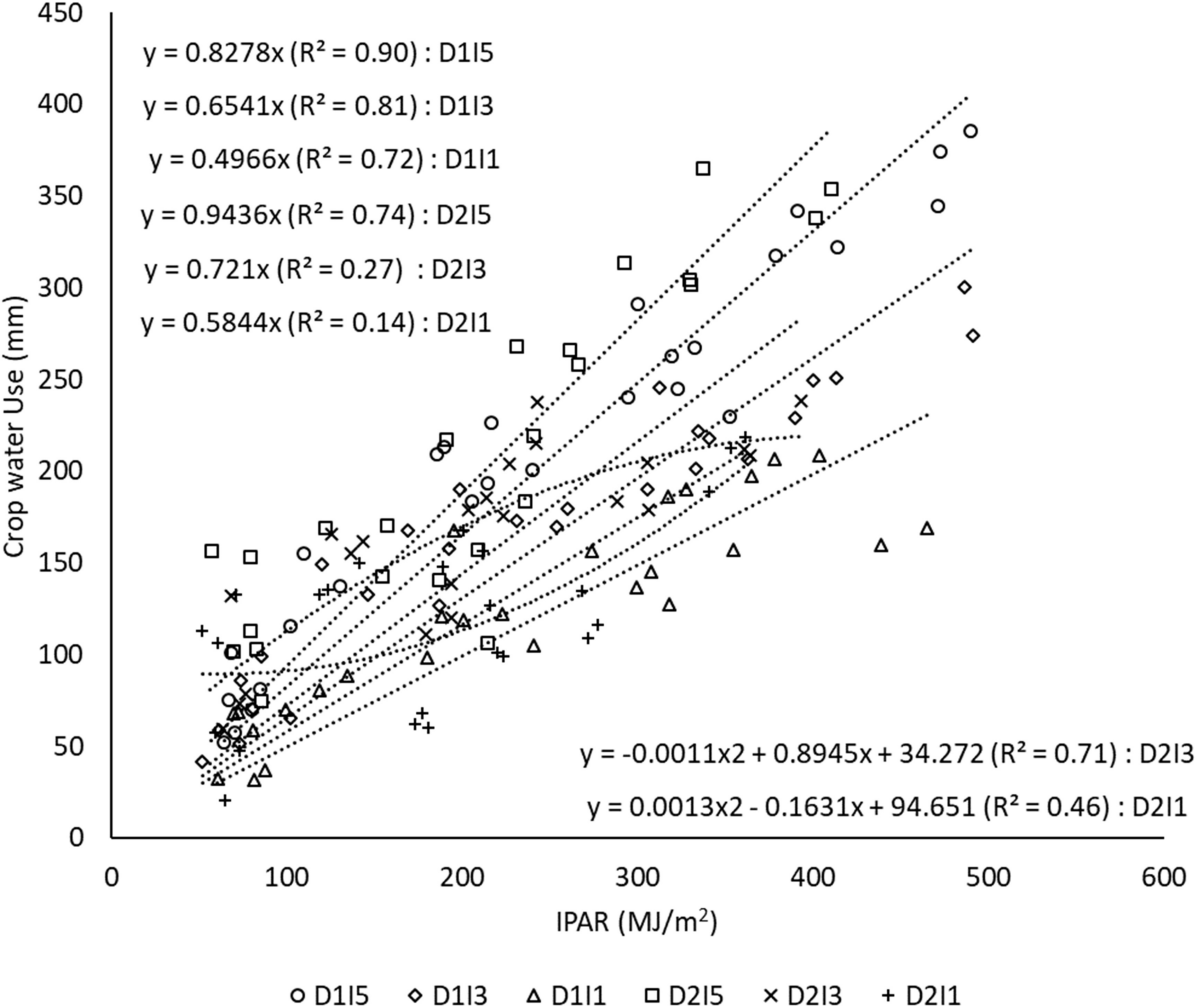
Regression between cumulative crop water use and cumulative intercepted PAR under different sowing and irrigation treatments (the slope indicates canopy conductance). Data pooled over two years. D1, normal sowing; D2 delayed sowing; I5, five irrigations; I3 three irrigations, and I1, one irrigation.

CC represents the amount of water crop uses per unit of radiation intercepted by the crop canopy during the crop growth. It is calculated as the slope of the regression line between cumulative crop water use and cumulative IPAR as shown in **Figure 10**. A delay in sowing caused an increase in the CC. The linear proportionality of crop water use per unit of IPAR was diminished to a greater extent under stressful environments due to delayed sowing and reduced water availability as exhibited by lower *R* ^2^ in D2I3 (0.27) and D2I1 (0.14). Under delayed sowing and reduced water availability conditions, the crop showed a non-linear (quadratic) response between crop water use and IPAR. It is supported by a higher *R* ^2^ of 0.71 and 0.46 for quadratic response function under D2I3 and D2I1 treatments, respectively. Even lowering of *R* ^2^ in D2I1 than in D2I3 further suggests that as the degree of stress increases, the interaction of thermal and water stress becomes more complex and even quadratic response may also not hold true in such cases.

### 3.8 Relation of yield and biomass with WP and RP

**Figure 11:**
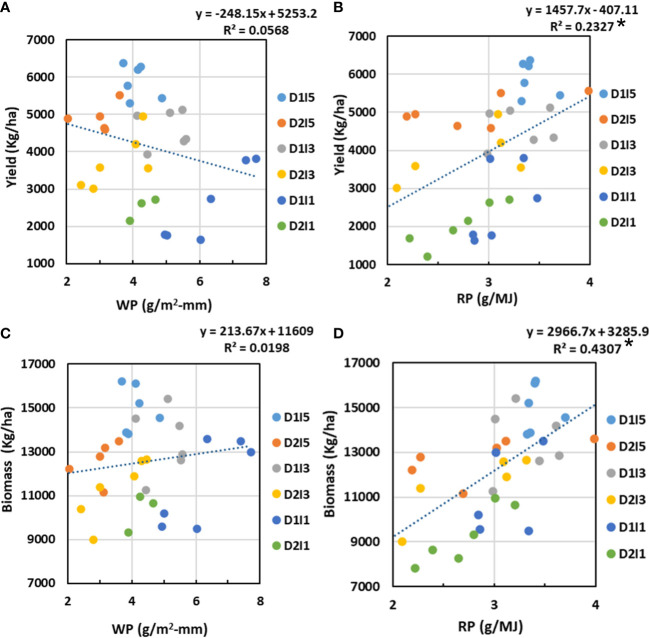
Relation between **(A)** WP and economic yield, **(B)** RP and economic yield, **(C)** WP and biomass, and **(D)** RP and biomass. D1, normal sowing; D2 delayed sowing; I5, five irrigations; I3 three inigations, and I1, one irrigation. Asterisk(*) indicates statistically significance of R^2^ at p<0.05.

The relation of resources (water and radiation) productivity with biomass and economic yield at harvest was analyzed through regression analysis, and its results are presented in **Figure 11**. It was observed that WP had no definite linear relation with final biomass or yield, as evidenced by poor *R* ^2^ values, which were statistically non-significant. However, RP had positive linear relation with both biomass and yield, but the relation was statistically significant with biomass (*R* ^2^ = 0.43, *p*< 0.05) but was non-significant with economic yield.

### 3.9 Coordination between WP and RP

**Figure 12:**
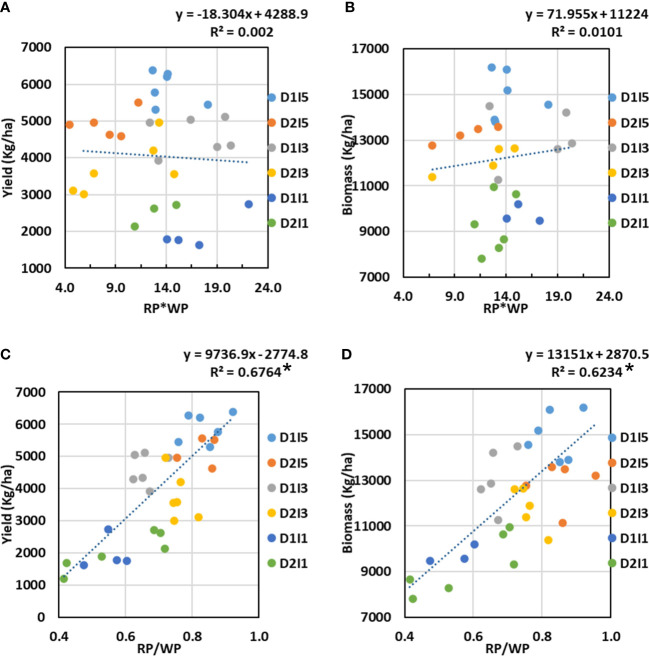
Relationship between **(A)** Yield versus the product of radiation productivity (RP) and water productivity (WP), **(B)** Biomass versus the product of RP and WP, **(C)** Yield versus a ratio of RP to WP, and **(D)** Biomass versus a ratio of RP to WP. D1, normal sowing; D2 delayed sowing; I5, five irrigations; I3 three inigations, and I1, one irrigation. Asterisk (*) indicates statistically significance of R^2^ at p<0.05.

We hypothesized that, independently, WP and RP may have poor relationships with final biomass/economic yield due to the interaction of multiple stresses to a metrics of coordination between WP and RP, which could better explain the variability in final biomass or yield. In order to explore the coordination of WP and RP, the two metrics tried were (a) the product of WP and RP (WP*RP) and (b) the ratio of RP and WP (RP/WP). These two metrics were regressed against the final biomass and yield for the treatments in 2 years. The results showed that WP*RP has no relation with final biomass or yield; however, the RP/WP ratio showed a significantly strong positive linear relationship with both final biomass and yield (**Figure 12**). The divisive effect of WP and RP on biomass/yield was much stronger than that of the multiplicative effect of either WP or RP alone. It can also be seen very clearly from **Figure 12** that the higher ratio of RP and WP, approaching unity, is associated with the higher biomass/yield under multiple stressed environments.

### 3.10 Relation between water and radiation productivity

**Figure 13:**
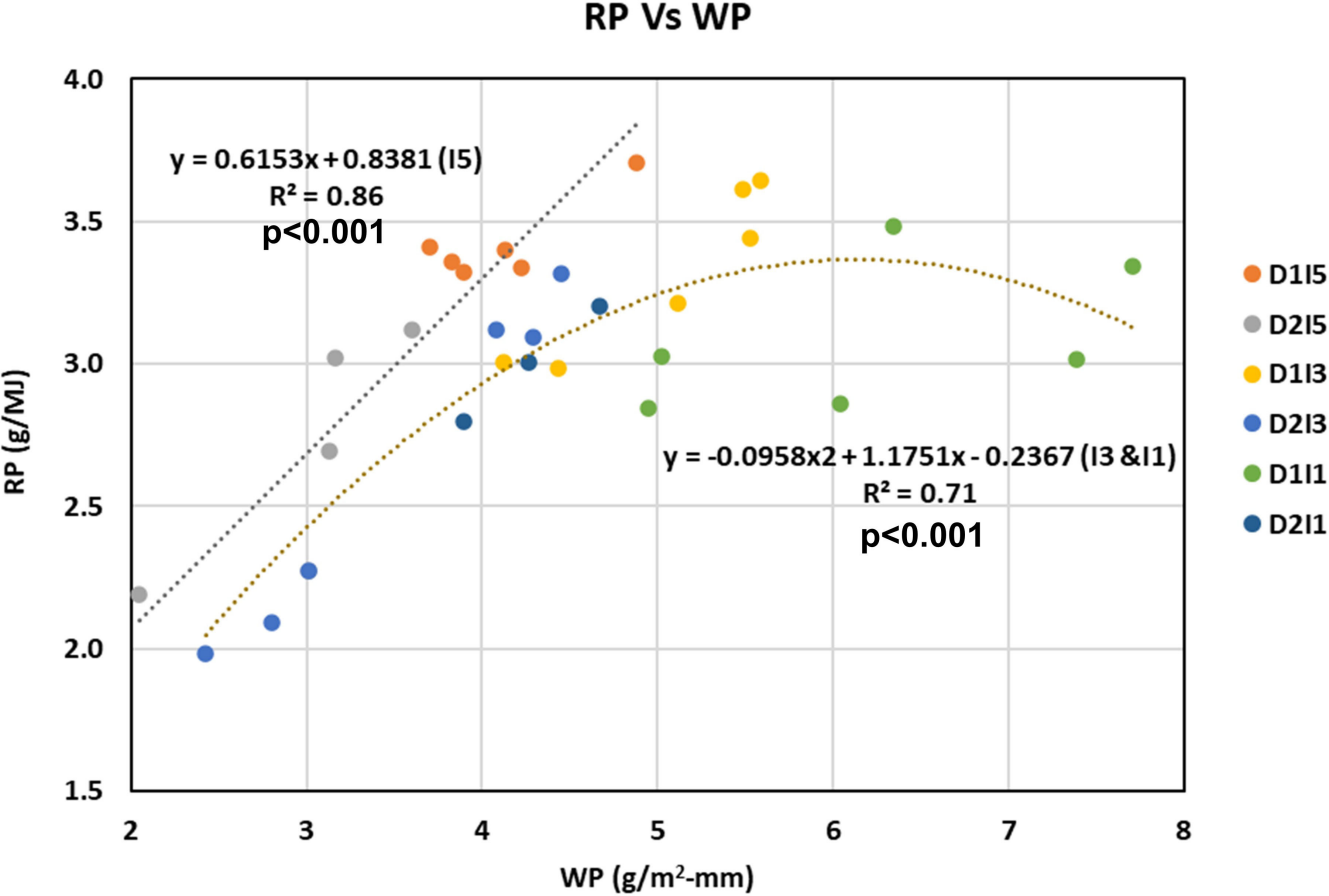
Understanding relation between WP and RP under non-stressed (15 treatments) versus stressed environments (I3 and I1 treatments). D1, normal sowing; D2 delayed sowing; I5, five irrigations; I3 three irrigations, and I1, one irrigation. Coefficient of determination (R^2^ is statistically significant at p<0.001.

To understand the association between WP and RP, the WP and RP under different sowing dates and irrigation treatments were plotted as a scatter diagram (**Figure 13**). It was observed that the linear proportionality of WP and RP holds true under non-stressful environments only (i.e., I5). Under stress conditions (I3 and I1), the linear proportionality of WP and RP is no longer valid, rather it follows a quadratic response function. It implies a trade-off between WP and RP under stressed conditions. **Figure 13** also shows that till mild levels of water stress, the increase in WP is accompanied by an increase in RP, but as water stress increases further, the RP stagnates, and as water stress levels increase further, the RP starts to decrease.

### 3.11 Yield penalty

**Figure 14:**
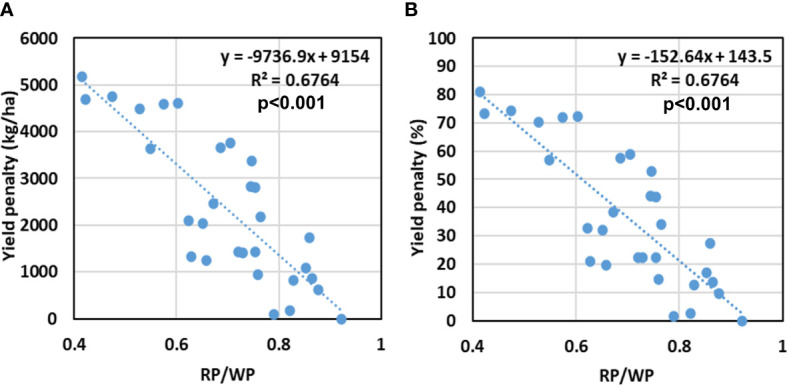
Relation between RP/WP to **(A)** absolute yield penalty (in kg/ha), and **(B)** percent yield penalty (%) for different treatments of sowing date and irrigation. Coefficient of determination (R^2^) is statistically significant at p<0.001.

**Figure 14** shows the relation between crop yield penalty and the ratio of RP to WP. The yield penalty here refers to the difference in economic yield realized (kg ha^−1^) in a treatment from the maximum economic yield obtained in any of the treatments in a crop season (**Figure 14A**). It was also expressed in terms of percentage (**Figure 14B**). There is a very significant inverse linear relation between the two. The increase in RP/WP ratio leads to a decrease in yield penalty under stresses. A 50% yield penalty was observed when the ratio of RP/WP reached approximately 0.61. It implies that under stress conditions, the yield penalty can be minimized by increasing both the productivities proportionally. A larger trade-off between the two productivities may result in a higher yield penalty.

## 4 Discussion

WP can be largely enhanced by increased production of biomass with no changes in crop water use or no change in biomass production but using less water or a combination of both (Blum, 2005). Analogously, improvement in RP can be achieved by manipulation of biomass production and IPAR. Understanding the behavior of crops for their biomass production, crop water use IPAR, and ultimately their resource use efficiency for water and radiation under stressful environments in field conditions is important. Field studies on WP and RP are challenging due to the lack of simple measurement techniques and the complexity of these traits (Hall et al., 1990; Sinclair and Muchow, 1999; Narayanan et al., 2013). The combined effect of water deficit and terminal heat stress on resource use efficiency (water and radiation) of field crops has not been well understood (Prasad et al., 2008a; Fahad et al., 2017) and is less researched. Water stress often limits leaf expansion, which is considered one of the most sensitive growth processes hit by drought (Alves and Setter, 2004). Lower seasonal crop water use under deficit irrigation conditions may be attributed to smaller transpiring surfaces coupled with low soil water availability even though large vapor pressure deficit conditions may exist during the growing season. The smaller differences in mean seasonal crop water use observed between normal sown and late sown crops may be ascribed to a lesser change in leaf elongation rate caused by terminal heat stress (Bos et al., 2000). Combined water and terminal heat stresses have an additive effect on crop water use.

A higher IPAR is more often associated with higher LAI (Han et al., 2008; Bassu et al., 2011). Higher IPAR by normal sown crop compared to late sown may be because of longer crop duration and higher LAI. The results were in conformity with the study by Pradhan et al. (2018). Irrigation treatments had a non-significant effect on seasonal IPAR, which may be attributed to the non-significant effect of irrigation treatment on plant architecture light extinction coefficient as reported by Thomas (2013) and Pradhan et al. (2018). Our results showed a non-significant effect of irrigation treatments on the light extinction coefficient. However, delayed sowing caused a moderate increase in the light extinction coefficient from 0.45 to 0.53. The delayed sowing resulted in shorter inter-nodal length, consequently decreasing plant height. This decrease in the vertical separation of leaves and reduced LAI resulted in changed canopy architecture, causing an increase in the light extinction coefficient. The combined effect of water and terminal heat stress had differential effects on LAI expansion and canopy architecture, thus causing a reduction in seasonal IPAR.

A decrease in the aboveground dry biomass of wheat under water stress conditions led to reduced leaf area and crop growth as a consequence of the reduction in cell division and elongation (Hussain et al., 2008; Farooq et al., 2009). The water deficit stress restricts the potential carbon assimilation rate mainly due to a reduction in leaf expansion, impaired photosynthetic apparatus, and early leaf senescence (Wahid et al., 2007). Another reason for low dry matter production under water stress is that it might have altered the photo-assimilate partitioning; more assimilate might have diverted toward roots under water stress conditions (Anapalli et al., 2008; Dhakar et al., 2018). The decrease in dry biomass of wheat under terminal heat stress conditions may be due to the shorter duration of grain filling (Prasad et al., 2008a, Prasad et al., 2008b), decreased photosynthesis (Djanaguiraman et al., 2020), and increased maintenance respiration. The combined effect of water and terminal heat stress on dry matter production was much higher than that of each stress alone, which resulted in a higher reduction in both IPAR and crop water use. Similar results of combined water and heat stress on dry biomass and yield have been reported on wheat and other crops (Wahid et al., 2007; Pradhan et al., 2012a; Lipiec et al., 2013; Sehgal et al., 2017).

The WP values obtained from the 2-year study (2.9 to 6.0 g m^−2^-mm) were within the range of those commonly reported for wheat (Caviglia and Sadras, 2001). The increase of WP under deficit irrigation conditions may be attributed to the well-known effect of stomatal closure without impairing the metabolic changes in the plant (Johnson, 1993; Morgan et al., 1993; Van den Boogaard et al., 1997; Rekika et al., 1998), but this increase in WP was at the cost of yield penalty. The doubling of WP (3 g m^−2^-mm to 6 g m^−2^-mm) caused the proportional yield penalty (approximately 5.0 t ha^−1^ to 2.5 t ha^−1^) under stresses (data not shown). A decrease in WP of wheat exposed to terminal heat stress may be associated with poor plant vigor, higher soil evaporation, and ultimately higher crop water use and a decrease in biomass. Early planting as an agronomic strategy has been suggested to have early crop vigor associated with higher WP (Oweis et al., 2000; Richards et al., 2002). However, the combined effect of water and terminal heat stress resulted in a decrease in WP. Though water stress leads to an increase in WP, severe water stress is also known to decrease WP (El Hafid et al., 1998; Anyia and Herzog, 2004). In our study, the additive effect of water and terminal heat stresses might have caused severe stress, which usually result in a decrease in photosynthesis due to decreased chlorophyll index, the quantum yield of the photosystem, and increased production of reactive oxygen species, and oxidative damage to membranes (Pradhan et al., 2012a; Pradhan et al., 2012b; Pradhan et al., 2012c; Pradhan and Prasad, 2015; Narayanan et al., 2016), consequently decreasing WP under combined water and heat stress (Lawlor, 2002).

It was observed that terminal heat stress in wheat caused a significant reduction in RP. This may be caused by a higher rate of reduction in biomass as compared to that of IPAR under delayed sowing. The higher rate of reduction in biomass in delayed sowing may be caused by higher maintenance respiration due to increased temperature as well as a decrease in grain filling duration. Irrigation treatments had a non-significant impact on RP, and these results are in conformity with other studies (Pradhan et al., 2018). RP and IPAR were more sensitive to high-temperature stress during the reproductive stage than water stress, as seen by the non-significant effect of irrigation treatments on RP and IPAR. Combined water and heat stress had a non-significant effect on RP, implying the domination of the non-significant effect of water stress on IPAR over the significance of terminal heat stress on IPAR. The values of RP (2.66 to 3.42 g/MJ) for wheat reported in this study were also consistent with values reported in earlier studies (Sinclair and Muchow, 1999; Pradhan et al., 2018).

CC may be taken as an indicator of aggregated leaf stomatal conductance at the canopy scale when the soil evaporation component is much lower (Sadras et al., 1991; Caviglia and Sadras, 2001). Caviglia and Sadras (2001) showed that the CC of wheat crop is largely unaltered by the contrasting supply of nitrogen in temperate environments. However, we found that CC is more affected by water stress than terminal heat stress in a subtropical environment. To the best of our knowledge, no study in the literature analyzed the behavior of crop conductance under combined water deficit and terminal heat stress. Our results showed that CC decreased significantly under deficit irrigation treatments, which is in conformity with the results of decreasing stomatal conductance under water stress in wheat (Nagar et al., 2015a). In contrast, the CC increased with the delay in sowing in our case. Literature also suggests an increase in stomatal conductance and decreased chlorophyll index under high temperature/heat stress (Pradhan et al., 2012a; Nagar et al., 2015b; Pradhan and Prasad, 2015). A decrease in stomatal conductance and chlorophyll index because of water deficit could also be a main reason for the reduced CO_2_ assimilation rate, which leads to lower grain yields.

Our study clearly showed a poor correlation of WP with final biomass and yield when data are pooled over the water and terminal heat stress treatments. These point to a complex interaction of water and heat stress on WP. Various studies have also reported no or poor definite relationship between transpiration efficiency, i.e., WP at the leaf level and yield (Specht et al., 2001; Saranga et al., 2004; Monneveux et al., 2005). However, RP was somewhat better related to final biomass and economic yield but the correlation values were still poor. It may be concluded that RP-based yield/biomass models may work better than that based on WP under a combined stress environment. Our hypothesis that coordination between RP and WP can better explain the variability in biomass/yield under multiple stresses than individually by RP or WP seems to hold true. Our field study clearly showed that the ratio of RP to WP is a much better metric to explain the variability in final yield/biomass than their product under multiple stress conditions. The highest biomass or yield is realized when the ratio of RP to WP approaches unity. We did not come across any such study in literature that depicted this kind of relationship at the field scale.

By definition, the ratio of RP to WP is mathematically equivalent to the ratio of crop water use to IPAR, i.e., CC. Thus, RP is expected to be linearly related to WP (Narayanan et al., 2013). However, our study found this positive linear relationship to hold under a non-stressful environment only. Stresses altered this relationship as both RP to WP and water use to IPAR (i.e., CC) (**Figures 10**, **13)** showed strong curvilinear relationships, implying a trade-off between the RP and WP under a stress environment. It can be seen that lower wheat yield is realized when we maximize or minimize both WP and RP. Lower yield under lower water or radiation productivities may be because of impairment of photosynthetic or metabolic machinery due to severe stress. Thus, it implies that coordination is required between these two productivities so as to maximize the yield gain or minimize the yield penalty under multiple stresses. However, it is important to confirm this with the range of wheat genotypes.

The suitability of WP as an important trait in breeding programs of crops under water-limited environments has been debated much but literature suggests that WP is a very complex trait and also not universal (Blum, 2005; Tambussi et al., 2007). Identifying traits that contribute to high yield potential under high temperature and water deficit stress is the prerequisite for a successful breeding program. It is suggested that screening for traits leading to a higher ratio of RP to WP for the selection of lines in wheat breeding may have scope to improve the wheat yield under multiple stress environments.

## 5 Conclusions

A 2-year field study was conducted on a dominant wheat cultivar (HD-2967) to quantify the interactive effect of water stress and terminal heat stress on growth, biomass, yield, and productivity of water and radiation. Normally, under a non-stress environment, the RP and WP are linearly related in wheat but the study showed that under the combined effect of increasing water stress and terminal heat stress, the linear proportionality between RP and WP breaks down. It implies that a trade-off happens between radiation and WP under an increasingly stressed environment and so there may be a coordinated response between the two. Furthermore, the study found that the ratio of RP to WP is a much better indicator of biomass and economical yield under combined stresses and both of these crop attributes can be maximized when this ratio approaches unity. The study concludes that breeders should identify traits in wheat plants that can help in maximizing the ratio of RP to WP to minimize yield penalty due to multiple stresses than looking at traits for increasing WP or RP separately. Selection of parents in wheat breeding based on such type of trait, i.e., ratio of RP and WP, may have a scope to improve yield under a stressed environment. It is expected that this behavior shall hold true for other wheat cultivars as well as for crops of the same family, in general. However, these results need to be further confirmed across different crops and their genotypes in different regions with varying degrees of multiple stresses and environments.

## Data availability statement

The raw data supporting the conclusions of this article will be made available by the authors, without undue reservation.

## Author contributions

RD and SN contributed equally and shared first authorship. RD, SN, VS designed the experiment, carried out the data analysis and prepared draft manuscript. RD, SN, VS, PJ, MS, DC, JM and PP interpreted the results and edited the manuscript. All authors contributed to the article and approved the submitted version.
